# Journeys into the genome of cancer cells

**DOI:** 10.1002/emmm.201202388

**Published:** 2013-01-22

**Authors:** Michael R Stratton

**Affiliations:** Wellcome Trust Sanger Institute, Wellcome Trust Genome CampusHinxton, UK

I come from a family in which there have been no scientists or doctors. I was interested, however, in biology at school and started my scientific career by training in medicine at Oxford University and Guys Hospital, London. Practising as a doctor reinforced my curiosity about the biological processes underlying human disease. As a consequence, I pursued a clinical vocation in histopathology, a discipline that couples exposure to the sights and smells of the autopsy room with a daily journey into the often beautiful, sometimes ugly world of healthy and diseased human tissues under the microscope. After an introduction to general histopathology in Nick Wright's department at the Hammersmith Hospital, London, I completed my postgraduate medical training in neuropathology with Peter Lantos at the Maudsley Hospital, London.

»Peering at the nuclei of cancer cells under the microscope, for me it was a matter of fascination that hidden within them were the key events converting normal cells into cancer cells, and frustration because they were out of reach.«

Many of the tissue samples examined by pathologists are from cancers. The clonal theory of cancer development and the general role of DNA mutations in generating cancer cell clones had been established by 1986 when I was working as a junior pathologist. Indeed, the first mutated cancer gene, *HRAS*, had recently been identified through application of the, then new, technologies of recombinant DNA technology. Peering at the nuclei of cancer cells under the microscope, for me it was a matter of fascination that hidden within them were the key events converting normal cells into cancer cells, and frustration because they were out of reach.

So, I took 3 years break from medicine to study for a PhD, learning the methods and thinking of molecular oncology in Colin Cooper's laboratory at the Institute of Cancer Research, London. Many Southern blots later I had experienced the joys and insights brought by detecting abnormalities of DNA in cancer cells. Essentially similar experiments characterize the science I have done since, at increasing scale, using different strategies and technologies, but with the same underlying aim.

## On the trail of cancer susceptibility genes

Having accredited as a pathologist, I returned to the Institute of Cancer Research as a Group Leader, with the intent of developing studies on the genetics of breast cancer susceptibility in collaboration with epidemiologists Doug Easton and Julian Peto. It had been recognized for many years that breast cancer cases clustered in some families. When I started, the field had just been set alight by the discovery, through genetic linkage analysis of such families, of the genomic location of the first high risk (10- to 20-fold) breast cancer susceptibility gene, *BRCA1*, on chromosome 17q. This discovery was a magnet for many of the major international groups in human disease genetics to engage in a highly publicised cloning race typical of the era to identify *BRCA1*. My fledgling team was certainly not in that league, and anyway, the job was clearly being done. Instead, I decided to explore the possibility that a further high-risk breast cancer susceptibility gene existed, although the evidence for this was by no means definitive. Nevertheless, after a couple of years acquiring breast cancer families that did not seem to be due to *BRCA1*, Richard Wooster (then a post doc with me) and the group embarked on another genome-wide search by genetic linkage analysis (in collaboration with David Goldgar and others) and ultimately located *BRCA2* in 1994 to chromosome 13q, in the process of course, also proving that the second gene existed (Wooster et al, 1994).

The next stage was to identify *BRCA2* itself. In this undertaking, we ourselves were now ensnared in a cloning race because we disagreed with the gene patenting and monopolization policies of Myriad Genetics, a biotechnology company from Utah, who became our competitors. Although the outlook initially did not look optimistic, we forged a collaboration with many participants, notably including Alan Ashworth, Andy Futreal and David Bentley (at the then Sanger Centre) and won the race at the end of 1995 (Wooster et al, 1995). Since then, analysis of *BRCA2* has entered routine clinical genetics practice to diagnose women at high risk of developing breast cancer.

Following the identification of *BRCA2*, we set off on a search for yet another high risk breast cancer gene, ‘*BRCA3*’. Unfortunately, this transpired to be a fruitless endeavour and the general conclusion now is that only two such genes exist. Nevertheless, a few years later with Nazneen Rahman, my erstwhile PhD student who was now leading the breast cancer genetics group at the Institute of Cancer Research, we started systematically sequencing candidate genes that were part of the DNA damage and repair pathways that include BRCA1/2 and identified a number of intermediate risk (two- to fourfold) breast cancer susceptibility genes including *CHEK2*, *ATM*, *BRIP1* and *PALB2*.

Towards the end of the last millennium, it was becoming apparent that most high-risk cancer susceptibility genes had been found. The era of genome-wide association studies to look for low risk susceptibility alleles was not yet upon us. Anyway, to me it felt like that enterprise would be more the domain of statisticians and epidemiologists than scientists with my background.

## Entering the uncharted jungle of cancer DNA

All cancers, however, are thought to arise through somatically acquired mutations. During the course of 1998–1999, in discussions with Richard Wooster and then Andy Futreal, the notion developed of using the reference human genome sequence, which was rapidly emerging from sequencing machines around the world, as a template against which we could sequence genome-wide for somatic mutations in cancer. It was clear that sequencing technology was not yet fit-for-purpose to do this, but perhaps the time had come to make a start.

Whichever way one looked at it, this experiment was big, full of risks, largely unpiloted and expensive. Not the sort of description that finds favour with most funding bodies. The Wellcome Trust, however, has a different approach to such matters. The Trust was already funding the sequencing of one-third of the reference human genome sequence and is accustomed to, indeed, has an appetite for, large-scale expeditions into the scientific darkness. Our Cancer Genome Project was approved for funding and started work in 2000 at the Wellcome Trust's genome facility, the Sanger Institute, near Cambridge, UK.

We started by sequencing coding exons in cancer genomes through PCR amplification and conventional Sanger sequencing. The amounts of DNA required were substantial, so we had to restrict ourselves to cancer cell lines, from which large quantities could be made. Unfortunately, very few of these lines had the available normal tissue DNA sample from the same individual that was necessary for us to call somatic mutations. By necessity, therefore, we embarked using a somewhat *ad hoc* assortment of 20–30 cancers, a few breast, a few lung, a few melanoma and odd examples of additional types. With the available technology, we were unable to plough through large numbers of genes and therefore assembled a shortlist with which to start. These genes were from pathways in which a gene was already known to be mutated and implicated in cancer development and/or genes encoding protein kinases, since the recent success of the targeted drug imatinib against the rearranged BCR-ABL protein in chronic myeloid leukaemia dramatically demonstrated how mutated kinases could be tractable drug targets.

## Occasional jewels among the sands of random mutations

No more than a few weeks into this early screen we began to see somatic mutations in the *BRAF* gene. *BRAF* encodes a cytoplasmic serine threonine protein kinase that is part of the well-studied RAS-RAF-MEK-ERK-MAP kinase signalling pathway. Further work demonstrated that *BRAF* mutations are present in approximately 60% malignant melanoma, 15% colorectal cancer, 30% papillary thyroid cancer and others. Biological studies conducted by Richard Marais and Chris Marshall confirmed that the mutations usually activate the BRAF kinase conferring transforming activity upon it (Davies et al, 2002).

Metastatic malignant melanoma is generally a remorseless disease unresponsive to conventional chemotherapy or radiotherapy. However, the nature of BRAF and its mutations recommended it as a drug target. In work conducted by others over the subsequent decade, small molecule inhibitors of mutated BRAF have been derived and shown to be effective in patients with metastatic malignant melanoma. Although these remarkable responses represent a major advance in treatment of the disease, resistant clones generally emerge and patients still succumb. Therefore, this is the beginning, rather than the end, of the story of a new approach to treating malignant melanoma (Flaherty et al, 2010).

**Figure 1:**
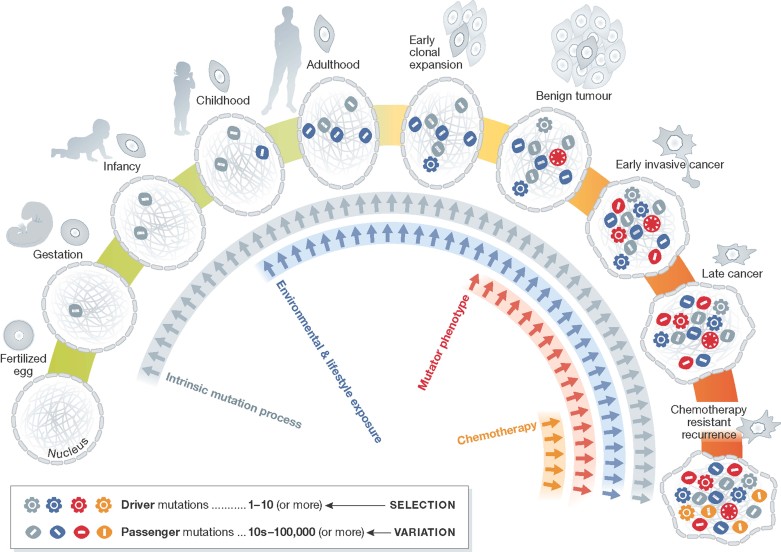
The cellular lineage between a fertilized egg and a fully malignant cancer cell Coloured symbols represent the accumulation of somatic mutations over a lifetime. The number of driver mutations reflects the number of biological processes that need to be subverted to convert a normal cell into a cancer cell. The genes in which driver mutations occur (cancer genes) can present tractable targets for new drug development. The number of passenger mutations reflects the number of mitoses between the fertilized egg and the cancer cell and the mutation rate at each mitosis. Passenger mutations provide insights into the underlying mutational processes operative in each case.

Meanwhile, back at the cancer genome, we were able to implement PCR-based conventional Sanger sequencing of exons to much higher throughput and apply it to primary cancer samples. This provided us with a bird's eye view of somatic mutations in cancer genomes. A small minority are ‘driver’ mutations in cancer genes, which convert normal cells into cancer cells, while the large majority are ‘passengers’ (Fig 1). A small number of cancer genes are mutated frequently, but many appear to contribute infrequently to cancer development (Greenman et al, 2007).

## The tangled and tattered strands of cancer DNA revealed

**Figure 2:**
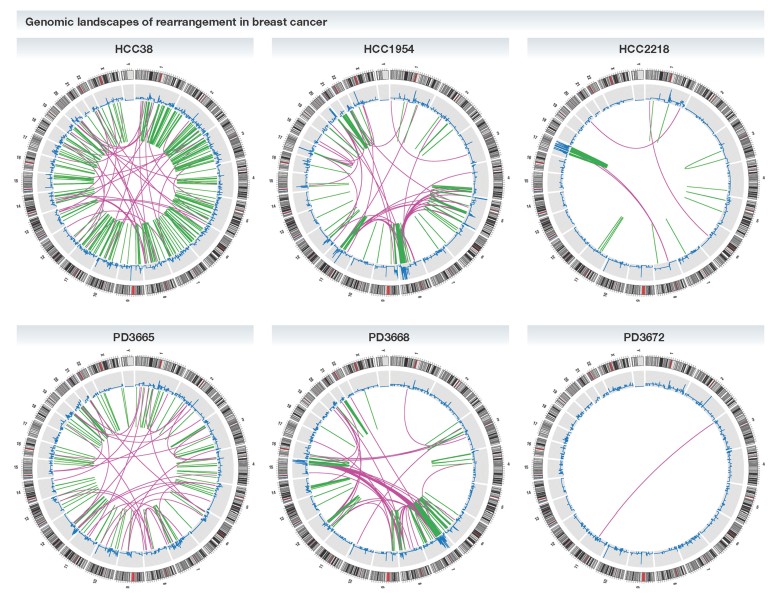
The genome-wide rearrangements in six breast cancer genomes (three cell lines, top and three primary tumours, below) In each case, the genome is represented as a circle and the lines represent somatic rearrangements, green are intrachromosomal and purple interchromosomal. First published in Stephens et al (2009).

The advent of next generation sequencing technologies around 2007 transformed our studies, and the field in general. Using these approaches, with Peter Campbell joining us at Sanger, we have been able to explore cancer genomes at sequence-level resolution revealing their extraordinarily contorted architecture (Fig 2; Stephens et al, 2009), producing essentially complete catalogues of somatic mutations from individual human cancers (Pleasance et al, 2010) and yielding many new mutated cancer genes (Stephens et al, 2012).

In an initiative reminiscent of the original Human Genome Project, with colleagues worldwide, the International Cancer Genome Consortium was constituted to coordinate the burgeoning sequencing activities across the range of human cancer types. Mutated in the appropriate manner, approximately 500 of the ∼20,000 protein coding genes in the human genome now appear to be causally implicated in the genesis of one or other of the 100–200 types of cancer. Given this diversity, enabling new drugs to be assessed, prior to starting clinical trials, for the cancer class and genome configuration that is most sensitive would be advantageous. With colleagues at the Massachusetts General Hospital, this has been set in train using 1000 genomically characterized cancer cell lines (Garnett et al, 2012).

Exploring cancer genomes continues to provide new intriguing dimensions of insight. Recently, we have shown that multiple underlying somatic mutational processes are operative in cancer, each of which can leave its own distinctive mutational signature on the genome (Nik-Zainal et al, 2012). Some may be due to exogenous exposures, others to abnormalities of DNA maintenance. Some operate genome-wide, others are targeted to small regions of the genome. Furthermore, it has allowed detailed analysis of the subclonal evolution of cancers, both within the primary cancer and in metastasis formation.

## Cancer genomes entering the clinic

Cancer genomics is already established in the clinical management of patients, as tests for mutations in certain genes, for example *EGFR*, *BRAF* and *HER2*, are required before drugs targeting the encoded protein can be prescribed. It is likely that this position will consolidate further and it is not unreasonable to speculate that in a decade whole cancer genome sequences may be routine for patients requiring cancer treatment. Over the next few years, the complete repertoire of mutated cancer genes across the spectrum of cancer types will be identified. Some are promising direct targets for new therapeutics and novel drugs are likely to emerge quickly. Others are not as tractable. However, they remain potential Achilles' heels of cancer cells and researchers will be exploring ways to somehow exploit them to develop new therapies. At the same time, deeper understanding of the mechanisms underlying the processes generating somatic mutations will lead to new insights into cancer causation and, potentially, new preventive strategies.
